# Involvement of Non-Coding RNAs in the Glucose Metabolic Reprogramming of Oral Squamous Cell Carcinoma: From Mechanisms to Therapeutics

**DOI:** 10.3390/biology15050373

**Published:** 2026-02-24

**Authors:** Jiaxin Huang, Minfei Liu, Ying Lin, Jiajun Mai, Jiashen Chen, Yiming Yang

**Affiliations:** Department of Basic Oral Medicine, School and Hospital of Stomatology, Guangdong Engineering Research Center of Oral Restoration and Reconstruction, Guangzhou Key Laboratory of Basic and Applied Research of Oral Regenerative Medicine, Guangzhou Medical University, Guangzhou 510182, China; jiaxinhuang0126@163.com (J.H.); fml171225@163.com (M.L.); ly2395759705@163.com (Y.L.); gmumaijiajun2022@163.com (J.M.); allen_yegel@163.com (J.C.)

**Keywords:** non-coding RNAs, oral squamous cell carcinoma, glucose metabolic reprogramming, glycolysis, biomarkers, therapeutic targets

## Abstract

Oral squamous cell carcinoma (OSCC) is a highly aggressive malignancy characterized by frequent recurrence and metastasis, which poses a significant global health problem. One of the prominent hallmarks of cancer is glucose metabolic reprogramming, wherein glycolysis is preferred over oxidative phosphorylation for macromolecule biosynthesis and energy production, even in the presence of oxygen. Non-coding RNAs (ncRNAs) are defined as a class of RNAs that are not translated into proteins, which include microRNAs, long non-coding RNAs, and circular RNAs. Recent studies have found that ncRNAs are crucial in regulating glycolysis in OSCC, wherein they reshape the metabolic landscape by modulating the expression of glucose transporters, essential enzymes, and transcription factors, ultimately influencing tumorigenesis. This comprehensive review systematically summarizes the regulatory mechanisms of ncRNAs involved in glucose metabolic reprogramming in OSCC, evaluates their potential as diagnostic biomarkers and therapeutic targets, and identifies clinically relevant ncRNAs through an integrative analysis of patient-derived data. These insights provide a mechanistic understanding of the metabolic alterations that drive progression in OSCC, as well as knowledge that can facilitate the development of clinically translatable targeted interventions for this aggressive malignancy.

## Simple Summary

Oral squamous cell carcinoma (OSCC) is an aggressive malignancy with a high risk of recurrence and metastasis. This review focuses on the roles of non-coding RNAs (ncRNAs) in driving OSCC progression through the modulation of glucose metabolic reprogramming. By systematically synthesizing evidence on ncRNA-mediated orchestration of key glycolytic regulators and incorporating OSCC patient-derived data, we identify several clinically relevant ncRNAs with potential utility as diagnostic biomarkers and therapeutic targets. These insights enhance our understanding of metabolic remodeling in OSCC and support the development of targeted, translationally relevant interventions to improve patient outcomes.

## 1. Introduction

Oral cancers, among which approximately 90% are oral squamous cell carcinoma (OSCC), heavily contribute to the global cancer burden [1,2]. In 2022, cancers of the lip and oral cavity accounted for 389,485 new cases and 188,230 deaths worldwide, ranking 16th and 15th out of 36 cancers in terms of prevalence and mortality rate, respectively [3]. The incidence of OSCC is projected to increase by 40% by 2040, highlighting the urgent need for improved prevention and treatment strategies [4]. The standard treatment for OSCC typically involves surgical resection with postoperative chemotherapy and radiotherapy, tailored to the tumor stage and individual patient characteristics. Unfortunately, more than half of patients with OSCC are diagnosed in advanced stages due to its high metastatic potential to regional lymph nodes and distant organs, including the lungs, bones, and liver, and thus OSCC is associated with poor prognosis and elevated recurrence rates [1,4]. Despite decades of medical advancements, the 5-year survival rates of OSCC remain suboptimal at 57.6% and 53.9% for stage III and IV, respectively [4]. Thus, research efforts for OSCC have intensified, specifically to develop more effective diagnostic and therapeutic approaches, with the ultimate goal of improving patient outcomes [5,6,7]. In recent years, modulating the cellular metabolic landscape has emerged as a promising target in the management of OSCC [8,9,10,11,12].

**Figure 1:**
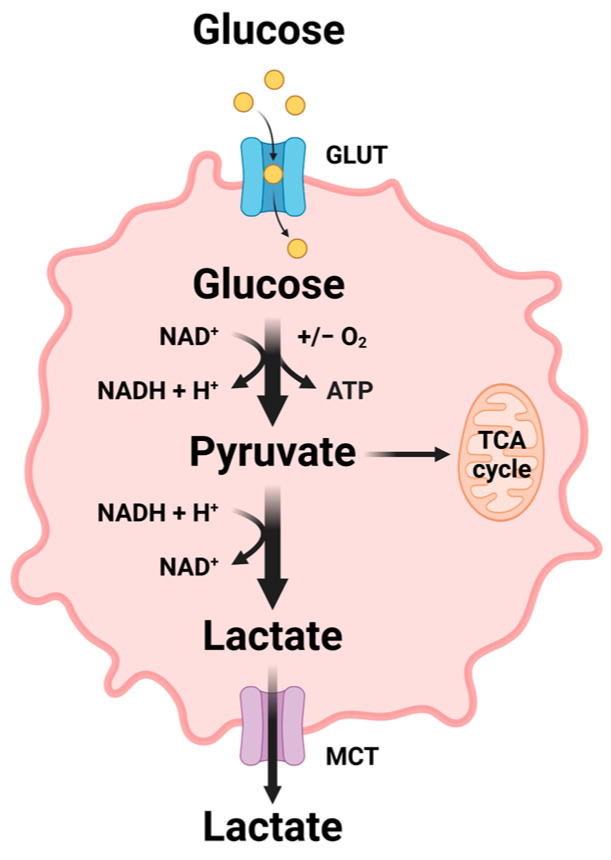
Aerobic glycolysis of tumor cells (the Warburg effect). GLUT, Glucose transporter; MCT, Monocarboxylate transporter; NAD^+^, Nicotinamide adenine dinucleotide; NADH, Nicotinamide adenine dinucleotide hydride; TCA, Tricarboxylic acid.

Metabolic reprogramming in cancer cells involves the adaptive transformation of metabolic phenotypes to support the requirements of rapid growth and proliferation [13]. Glucose, lipids, and amino acids are the major substances involved in cellular energy metabolism, with glucose being central to metabolic reprogramming. One hallmark of tumor cells is aerobic glycolysis (i.e., the Warburg effect), which is characterized by a preference for glycolysis and lactate secretion even under aerobic conditions (Figure 1). This metabolic rewiring is closely linked to the upregulation of glucose transporters (i.e., GLUT1 and GLUT3) and the aberrant activation of key glycolytic enzymes, leading to an abundant yield of metabolic intermediates used for massive biosynthesis and tumor progression [14,15,16]. Additionally, the pentose phosphate pathway (PPP), a branch pathway for glucose oxidation, generates nicotinamide adenine dinucleotide phosphate (NADPH) and nucleotide precursors that contribute toward tumor survival and growth [17]. Several clinical trials have demonstrated the anticancer potential of targeting the key metabolic regulators in these pathways, particularly by inhibiting GLUT and glycolytic enzymes [18,19].

Recent studies suggest that non-coding RNAs (ncRNAs) play a pivotal role in the regulation of glucose metabolic reprogramming in OSCC [20,21,22]. This regulatory mechanism is closely associated with tumor proliferation and metastasis, thereby greatly influencing patient outcomes. For instance, lnc-p23154 was found to be upregulated in OSCC tissues compared to peri-tumor tissues, promoting OSCC metastasis by enhancing GLUT1-mediated glycolysis [23]. Moreover, circBICD2, a regulator of the key glycolytic enzyme hexokinase 2 (HK2), has been identified as a candidate therapeutic target in OSCC in preclinical studies [24]. These findings highlight the great potential of ncRNAs in the discovery of novel diagnostic biomarkers and therapeutic targets, which can open new avenues for the treatment of OSCC. While previous reviews have provided valuable insights into ncRNAs in head and neck cancers [25,26,27] and their roles in metabolic reprogramming [22], an overview focusing specifically on the regulatory networks of distinct ncRNA classes in glucose metabolism in OSCC remains warranted.

To address this gap, the present review provides a comprehensive update on the regulatory roles of ncRNAs in glucose metabolic reprogramming in OSCC and explores their potential clinical relevance. Specifically, we focus on three major questions: how different ncRNA classes (microRNAs [miRNAs], long non-coding RNAs [lncRNAs], and circular RNAs [circRNAs]) regulate key components of glucose metabolism—including transporters, enzymes, and transcription factors—in OSCC; what clinical relevance these ncRNA-mediated metabolic axes hold as potential biomarkers or therapeutic targets; and whether OSCC-specific ncRNA networks exist that could be leveraged for precision medicine. By integrating a thorough synthesis of established mechanisms with original bioinformatic analyses of The Cancer Genome Atlas (TCGA)–OSCC data, we identify glycolysis-associated ncRNAs with prognostic value, and highlight prevailing conceptual and technical challenges. Collectively, this review advances the current understanding of ncRNA-driven metabolic reprogramming in OSCC and provides a framework for future mechanistic and translational research.

## 2. Classification and Functions of ncRNAs

By definition, ncRNAs are a diverse class of transcriptional products that are not translated into proteins [28]. Functionally, ncRNAs are categorized into housekeeping ncRNAs and regulatory ncRNAs. Housekeeping ncRNAs include ribosomal RNAs (rRNAs), transfer RNAs (tRNAs), small nuclear RNAs (snRNAs), and small nucleolar RNAs (snoRNAs). On the other hand, regulatory ncRNAs are further divided into small and long ncRNAs based on their length. Small ncRNAs include miRNAs, small interfering RNAs (siRNAs), and PIWI-interacting RNAs (piRNAs), whereas lncRNAs, typically exceeding 200 nt in length, include long intergenic ncRNAs (lincRNAs), circRNAs, and pseudogene-derived lncRNAs [28,29]. In particular, miRNAs, lncRNAs, and circRNAs have emerged as significant regulatory molecules.

**Figure 2:**
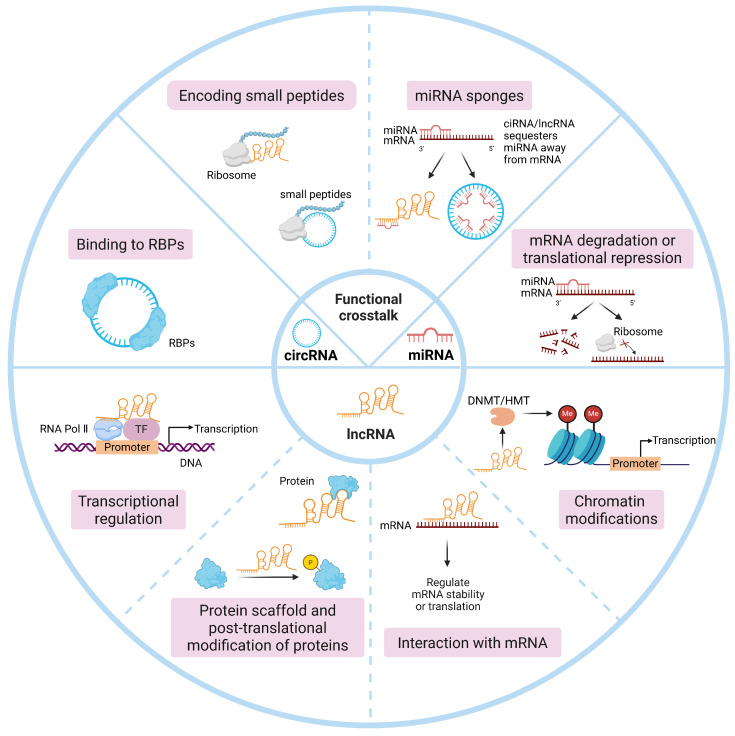
The main functions of miRNA, lncRNA, and circRNA. DNMT, DNA methyltransferases; HMT, Histone methyltransferases; RBPs, RNA-binding proteins; RNA Pol II, RNA polymerase II; TF, Transcription factor.

miRNAs, which are approximately 22 nt in length, regulate gene expression by binding to complementary sequences in the 3′ untranslated region (UTR) of target mRNAs. This binding triggers mRNA degradation or translational repression via the RNA-induced silencing complex (RISC) [30]. On the other hand, lncRNAs and circRNAs exhibit more diverse and intricate regulatory networks, with various interactions with DNA, RNA, and proteins. Their primary modes of action include acting as sponges for miRNAs and proteins, regulating transcription, serving as protein scaffolds, mediating chromatin remodeling and post-translational modifications of proteins, and even encoding small peptides (Figure 2). This remarkable functional versatility underscores their critical role in regulating various pathophysiological processes [30,31,32].

## 3. ncRNA-Mediated Glucose Metabolic Reprogramming in OSCC

**Figure 3:**
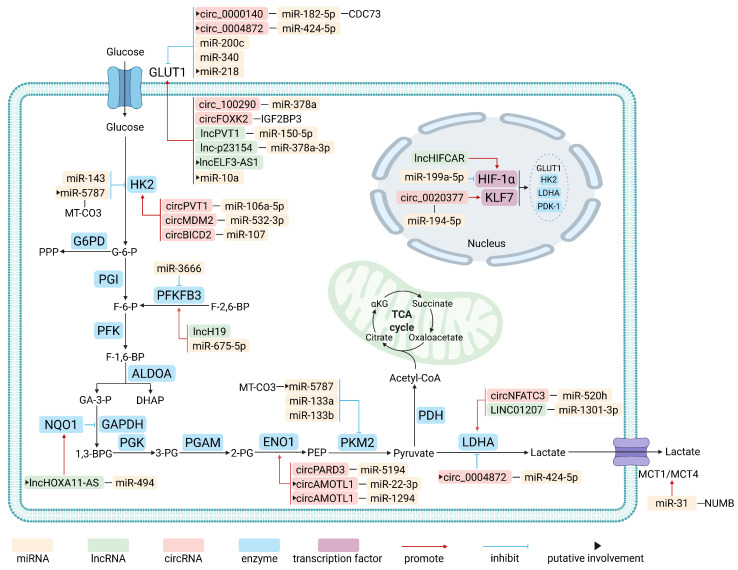
Regulatory roles of miRNAs, lncRNAs, and circRNAs in glucose metabolism in oral squamous cell carcinoma. 1,3-BPG, 1,3-Bisphosphoglycerate; 2-PG, 2-Phosphoglycerate; 3-PG, 3-Phosphoglycerate; αKG, α-Ketoglutarate; ALDOA, Aldolase A; CDC73, Cell division cycle 73; DHAP, Dihydroxyacetone phosphate; ENO1, Enolase1; F-1,6-BP, Fructose-1,6-bisphosphate; F-2,6-BP, Fructose-2,6-bisphosphate; F-6-P, Fructose-6-phosphate; G-6-P, Glucose-6-phosphate; G6PD, Glucose-6-phosphate dehydrogenase; GA-3-P, Glyceraldehyde-3-phosphate; GAPDH, Glyceraldehyde-3-phosphate dehydrogenase; GLUT1, Glucose transporter 1; HIF-1α, Hypoxia-inducible factor 1 alpha; HK2, Hexokinase 2; IGF2BP3, Insulin-like growth factor 2 mRNA binding protein 3; KLF7, Krüppel-like factor 7; LDHA, Lactate dehydrogenase A; MCT, Monocarboxylate transporter; MT-CO3, Mitochondrial cytochrome c oxidase subunit 3; NQO1, NAD(P)H quinone oxidoreductase 1; PDH, Pyruvate dehydrogenase; PDK-1, Pyruvate dehydrogenase kinase 1; PEP, Phosphoenolpyruvate; PFK, Phosphofructokinase; PFKFB3, 6-Phosphofructo-2-kinase/Fructose-2,6-bisphosphatase 3; PGAM, Phosphoglycerate mutase; PGI, Glucose-6-phosphate isomerase; PGK, Phosphoglycerate kinase; PKM2, Pyruvate kinase M2; PPP, Pentose phosphate pathway; TCA, Tricarboxylic acid.

Aerobic glycolysis in OSCC is a coordinated metabolic adaptation that fuels tumor growth and shapes the tumor microenvironment (TME) [33,34]. This process involves enhanced glucose uptake, accelerated glycolytic flux, and activation of master transcriptional regulators. Beyond providing ATP and biosynthetic precursors (e.g., nucleotides and lipids), aerobic glycolysis results in massive lactate secretion, acidifying the TME to promote immune evasion, invasion, and metastasis [35]. ncRNAs orchestrate tumor progression by modulating key components of glucose metabolism, including glucose transporters, metabolic enzymes, and transcription factors. A detailed overview of the ncRNAs involved in regulating glycolysis in OSCC is presented in Figure 3.

### 3.1. Regulation of Glucose Uptake by ncRNAs

Glucose metabolic reprogramming is substantially mediated through the modulation of GLUT, which are membrane proteins that facilitate glucose translocation from the bloodstream into the cells. Increased glucose uptake, mediated by the upregulation of GLUT expression, meets the high metabolic demand of proliferating cells. The GLUT family includes 14 members, among which GLUT1 and GLUT3 are the most extensively characterized [36]. In OSCC, various ncRNAs regulate GLUT1 expression through multiple mechanisms, thereby reprogramming glucose uptake and glycolysis.

First, the competing endogenous RNA (ceRNA) model represents the most extensively characterized mechanism. In this framework, specific ncRNAs act as molecular sponges that sequester miRNAs, thereby alleviating post-transcriptional repression of GLUT1 mRNA and promoting glycolytic flux. For example, circ_100290 [37] and lncPVT1 [38] have been experimentally validated through dual-luciferase reporter and rescue assays to sponge miR-378a and miR-150-5p, respectively, both of which directly target the 3′ UTR of GLUT1 mRNA. In contrast, other ncRNAs, such as circ_0004872 [39] and circGDI2 [40], have been proposed to modulate glucose metabolism via the miR-424-5p axis and may indirectly influence GLUT1 expression. However, the mechanistic evidence remains incomplete, as two critical interactions require confirmation: (1) direct binding of miR-424-5p to GLUT1 mRNA, and (2) physical interaction between these circRNAs and the miRNA. Future studies should prioritize direct experimental validation, such as dual-luciferase reporter assays and RNA immunoprecipitation (RIP), to definitively establish these functional links. Beyond these canonical cytoplasmic ceRNA interactions, the nucleus-enriched and OSCC-specific lnc-p23154 promotes glycolysis and metastatic behavior via the lnc-p23154/miR-378a-3p/GLUT1 axis [23]. Mechanistically, lnc-p23154 represses the transcription of miR-378a-3p, a microRNA that directly targets the 3′ UTR of GLUT1 mRNA, thereby augmenting GLUT1-mediated glucose metabolism. Importantly, lnc-p23154 has thus far been reported exclusively in OSCC, suggesting a tumor-type-specific role in metabolic control. Collectively, these findings establish the ceRNA network as a prevalent and versatile layer of GLUT1 regulation in OSCC while also highlighting the need for more rigorous functional validation to distinguish direct mechanistic interactions from indirect associations.

Second, certain ncRNAs regulate GLUT1 by directly interacting with its mRNA to enhance transcript stability or translation efficiency. For instance, lncELF3-AS1 is proposed to promote GLUT1 expression through such an interaction, a mechanism that awaits validation from RNA pull-down or RIP assays [41]. In the miRNA realm, the direct targeting and suppression of GLUT1 mRNA by miR-340 is well-established through luciferase assays [42], whereas the regulatory roles of miR-218 [43] and miR-10a [44] are supported only by correlative or bioinformatic evidence.

Third, N^6^-methyladenosine (m^6^A) modification regulates ncRNA functions in OSCC by altering their interactions with RNA-binding proteins. For example, Cui et al. demonstrated that m^6^A modification on circFOXK2 recruits the m^6^A reader protein insulin-like growth factor 2 mRNA binding protein 3 (IGF2BP3). The resulting circFOXK2/IGF2BP3 complex then recognizes and binds to m^6^A sites on target mRNAs such as GLUT1, thereby enhancing mRNA stability and expression to drive glycolysis [45]. This illustrates how m^6^A modification on an ncRNA confers new regulatory functions, allowing it to act as an adaptor to promote gene expression via this epitranscriptomic mechanism.

GLUT3, another important member of the GLUT family, is similarly regulated by ncRNAs, but this has only been documented in other types of head and neck squamous cell carcinoma (HNSCC) and not in OSCC. Wang et al. found that miR-23a-3p suppresses GLUT3 expression via the miR-23a-3p/sine oculis homeobox homolog 1 (SIX1)/GLUT3 axis, inhibiting glucose uptake and glycolysis in HNSCC as a result [46]. Current research on the ncRNA-mediated regulation of GLUTs in OSCC has predominantly focused on GLUT1. On the other hand, other GLUT isoforms remain largely unexplored, representing a significant knowledge gap in the regulatory network of GLUTs in OSCC. In summary, the regulatory influence of ncRNAs on GLUT3 and other non-GLUT1 glucose transporters remains an important, underexplored frontier in OSCC biology.

### 3.2. Regulation of Metabolic Enzymes by ncRNAs

An abundant amount of energy is required for malignant growth, proliferation, and metastasis. To sustain these processes, cancer cells preferentially utilize aerobic glycolysis to produce energy and metabolic intermediates, even in the presence of oxygen [47,48]. This typically involves the upregulation of metabolic enzymes, such as HK2, 6-phosphofructo-2-kinase/fructose-2,6-bisphosphatase (PFKFB), pyruvate kinase M2 (PKM2) and lactate dehydrogenase A (LDHA), to accelerate the rate of glycolysis [49]. Increasing evidence has indicated that ncRNAs can either promote or inhibit glycolysis in OSCC through the regulation of various glycolytic factors or key enzymes.

In the initial rate-limiting step of glycolysis, HK catalyzes the transformation of glucose into glucose-6-phosphate (G-6-P) [50], which is irreversible under physiological conditions [51]. In mammalian tissues, HK manifests in four isoforms, namely HK1, HK2, HK3, and HK4. Among these, HK2 stands out as the most significant subtype in cancer cells, and changes in its expression are important in modulating tumor initiation and progression [52]. In HNSCC, HK2 expression is significantly elevated [53]. The dysfunction of HK2 in OSCC is regulated by ncRNAs. Among the miRNAs, miR-143 functions as a tumor suppressor by directly targeting HK2, thus inhibiting cell migration, glucose metabolism, and proliferation [54]. Similar inhibitory effects of miR-143 on HK2-mediated glycolysis have also been reported in glioblastoma [55] and breast cancer [56]. While these findings suggest that the miR-143/HK2 regulatory axis may be conserved across various cancers, it is important to recognize that OSCC-specific factors, including tissue heterogeneity, the tumor microenvironment, and differential expression of related signaling pathways, could influence its functional outcomes. Therefore, although the miR-143/HK2 axis represents a potential therapeutic target, further investigation in the context of OSCC is essential to validate its clinical and translational relevance. Meanwhile, Chen et al. found that miR-5787 downregulation contributed to chemoresistance in tongue squamous cell carcinoma (TSCC) cells by targeting mitochondrial cytochrome c oxidase subunit 3 (MT-CO3) to suppress oxidative phosphorylation (OXPHOS), thus activating aerobic glycolysis by the upregulation of HK2 [57]. Sufficient evidence has shown that circRNAs can specifically sponge miRNAs, thereby functioning as ceRNAs and mediating tumor glucose metabolic reprogramming [58]. In OSCC, circPVT1 competitively binds miR-106a-5p, relieving its inhibitory effect on HK2 and thereby promoting glycolysis, proliferation, and metastasis [59]. Likewise, circBICD2 suppresses miR-107 to increase HK2 expression and enhance glycolytic activity [24], while the circMDM2/miR-532-3p/HK2 axis similarly promotes glycolysis and contributes to metabolic reprogramming in OSCC [20]. Given that the regulation of HK2 by circBICD2 and circMDM2 has so far been reported exclusively in OSCC, targeting these ceRNA pathways could, in principle, offer high tumor specificity. Further validation across other cancer types is necessary to confirm this proposed specificity. Therefore, HK2 serves as a critical nexus in OSCC glycolysis, tightly regulated by a diverse network of ncRNAs. This regulatory interplay presents both conserved and potentially OSCC-specific therapeutic vulnerabilities.

The PFKFB family includes bidirectional glycolytic enzymes that regulate the formation and degradation of fructose-2,6-bisphosphate (F-2,6-BP) [60]. Four distinct members have been identified [61]: (1) PFKFB1, found in the liver and skeletal muscle; (2) PFKFB2, predominantly expressed in the cardiac muscle; (3) PFKFB3, which is ubiquitously expressed; and (4) PFKFB4, mainly present in the testes [62]. In particular, PFKFB3 is highly expressed in proliferating normal tissues, such as the thymus, and in many cancer cells [63]. Mounting evidence suggests the potential of PFKFB3 as a target for anticancer therapy due to its role in regulating glycolysis in cancer cells. Yang et al. reported that lncH19 regulates glycolysis in cancer-associated fibroblasts via the lncH19/miR-675-5p/PFKFB3 axis, which was validated using a nude mouse xenograft tumor model [21]. In addition, Chen et al. confirmed that PFKFB3 is a direct target of miR-3666 in HNSCC and demonstrated through rescue assays that miR-3666 suppresses glycolysis and cell growth in the OSCC cell line Cal27 [64]. Aside from PFKFB3, PFKFB4 expression is also significantly elevated in OSCC, thus providing a fresh perspective on the metabolic characteristics of OSCC [60]. Certain ncRNAs are known to influence glucose metabolism by regulating PFKFB4 expression in other tumors [65,66], but similar reports in OSCC are scarce, positioning this as an area for future study. In conclusion, the PFKFB family represents a viable metabolic target in OSCC, with PFKFB3 regulation being partially characterized and PFKFB4 offering a novel avenue for investigation.

Glyceraldehyde-3-phosphate dehydrogenase (GAPDH) catalyzes the nicotinamide adenine dinucleotide (NAD)-dependent oxidation and phosphorylation of glyceraldehyde-3-phosphate (G-3-P) to 1,3-bisphosphoglycerate (1,3-BPG) [67]. The recently identified lncHOXA11-AS enhances NAD(P)H quinone oxidoreductase 1 (NQO1) expression by sponging miR-494 [68], thereby elevating NQO1 enzymatic activity and promoting the consumption of flavin adenine dinucleotide (FAD), a crucial coenzyme for GAPDH. The resulting FAD depletion may attenuate GAPDH activity and consequently suppress glycolysis in OSCC cells. However, direct rescue assays (e.g., GAPDH knockdown or overexpression) validating the causal involvement of FAD-mediated GAPDH regulation in lncHOXA11-AS/NQO1-driven glycolytic inhibition are still lacking. Despite the absence of experimental confirmation, this potential crosstalk between the lncHOXA11-AS/NQO1 axis and GAPDH provides an intriguing direction for further metabolic investigations in OSCC.

Enolase 1 (ENO1) catalyzes the dehydration of 2-phosphoglyceric acid to phosphoenolpyruvate (PEP) in glycolysis [69]. ENO1 is highly expressed in various tumors, such as hepatocellular carcinoma (HCC) [70], breast cancer [71], and gastric cancer [72]. In line with this, Liu et al. found that ENO1 expression was significantly increased in the OSCC cell line Cal27. Mechanistically, circAMOTL1 enhances glycolysis and OSCC cell proliferation by stabilizing ENO1 mRNA through sponging miR-22-3p and miR-1294 [73]. However, this only confirms an indirect regulatory relationship between miRNA and ENO1 through bioinformatics prediction and expression correlation. Future studies could apply luciferase reporter assays, RIP, or biotin-labeled RNA pull-down experiments to confirm this direct interaction. Nevertheless, Luo et al. discovered that circPARD3 plays an oncogenic role in OSCC through a novel circPARD3/miR-5194/ENO1 axis, making it a candidate therapeutic target [74]. These findings underscore the ability of circRNAs to modulate ENO1 expression, highlighting the need for further mechanistic validation to explore their therapeutic implications in OSCC.

The final step of glycolysis is catalyzed by pyruvate kinase (PK) through a transphosphorylation reaction between phosphoenolpyruvate (PEP) and adenosine diphosphate (ADP) [75]. Noguchi et al. showed that *PKM* encodes two subtypes of PK, PKM1 and PKM2 [76], which have different catalytic properties and tissue distribution despite their sequence similarity. PKM1, mainly expressed in energy-demanding tissues such as muscle and brain, forms a stable tetramer that efficiently drives glycolysis [77,78]. In contrast, PKM2 predominates in proliferative cells (embryonic cells, stem cells, and cancer cells) and can exist as either active tetramers or less active dimers [75]. The tetrameric form exhibits high catalytic activity that drives the rapid conversion of PEP to pyruvate, thereby restricting the availability of glycolytic intermediates for biosynthesis. Conversely, under nutrient limitation, PKM2 predominantly adopts a low-activity dimeric conformation, which slows pyruvate production and facilitates the accumulation of intermediates to fuel anabolic processes [79]. Given its pivotal role in metabolic reprogramming, PKM2 has emerged as a potential therapeutic target in cancer. In OSCC, miR-5787 knockdown increases PKM2 expression and enhances glycolytic activity [57], and miR-133a and miR-133b exert similar regulatory effects [80]. Thus, the structural plasticity of PKM2 acts as a pivotal metabolic switch in OSCC, and its regulation by specific miRNAs fine-tunes glycolytic flux to meet the biosynthetic demands of tumor cells.

LDHA catalyzes the reduction of pyruvate to lactate, concurrently oxidizing nicotinamide adenine dinucleotide hydride (NADH) back to NAD^+^ [81]. The regeneration of NAD^+^ is important because it is an essential coenzyme for the catalytic activity of the glycolytic enzyme GAPDH, and it ensures the continuous progression of the glycolysis pathway. Excess lactate causes the TME to become acidic, which accelerates cancer progression and metastases [82], positioning LDHA as a crucial target for cancer treatment. Moreover, the activity of LDHA is also regulated by ncRNAs. Xie et al. found that circNFATC3 and LDHA were upregulated, whereas miR-520h was downregulated in OSCC cells. Mechanistically, circNFATC3 sponges miR-520h to elevate LDHA expression, thereby promoting glycolytic metabolism and cell proliferation [83]. Similarly, LINC01207 promotes LDHA expression by competitively binding miR-1301-3p, thereby stimulating OSCC cell proliferation and migration while suppressing apoptosis and autophagy [84]. These studies focus primarily on cell-autonomous phenotypes, where ncRNA-mediated LDHA upregulation drives lactate overproduction. This process directly promotes TME acidification and metabolic reprogramming, thereby fueling tumor progression and metastasis. Additionally, ncRNAs can also function as tumor suppressor factors by downregulating the expression of certain enzymes [85,86]. For instance, circ_0004872 inhibits glycolysis by sequestering miR-424-5p, a known positive regulator of LDHA expression [39]. Given the opposing regulatory roles of different ncRNAs, combined modulation of multiple ncRNAs may more effectively inhibit LDHA activity and OSCC progression. Specifically, simultaneously inhibiting LDHA-promoting ncRNAs (e.g., circNFATC3, LINC01207) and activating LDHA-suppressing ones (e.g., circ_0004872) could yield synergistic anticancer effects. Future studies should further evaluate this multi-target regulatory network to identify optimal therapeutic combinations. Beyond regulating lactate production through LDHA, ncRNAs also influence lactate efflux by modulating monocarboxylate transporters (MCTs), which mediate lactate shuttling across the plasma membrane [87]. A prominent example in OSCC is the oncogenic miR-31, which directly targets the adaptor protein NUMB [88]. The consequent downregulation of NUMB increases the stability and expression of MCT1 and MCT4, thereby enhancing lactate production and export to ultimately promote tumor acidosis and progression. Targeting ncRNA-mediated regulation of LDHA and MCTs represents a viable strategy to inhibit lactate production and tumor progression in OSCC.

Previous studies exploring the ability of ncRNAs to regulate key enzymes mainly focused on enzyme expression rather than on the regulation of enzyme activity. Enzyme activity refers to the ability of an enzyme to catalyze a reaction, which is usually measured using a specific substrate conversion rate. Changes in enzyme activity directly indicate the metabolic status of a cell and its pathological changes. On the other hand, enzyme expression is typically measured based on protein concentration or mRNA expression levels [89]. It is important to note that an increased enzyme expression does not necessarily correlate with increased enzyme activity because a variety of factors influence the latter, such as post-translational modifications, substrate availability, and the presence of inhibitors [90,91]. Thus, measuring enzyme expression alone is insufficient to fully elucidate the regulatory role of ncRNAs on key glucose metabolic enzymes. Future studies should integrate the analysis of both enzyme activity and expression to better understand ncRNA-mediated regulatory mechanisms and their roles in disease initiation and progression.

### 3.3. Regulation of Transcription Factors by ncRNAs

Glucose metabolism in OSCC is partly regulated by ncRNAs through their influence on transcription factors. For example, the transcription factor hypoxia-inducible factor 1 alpha (HIF-1α) is a key regulator of glycolysis, and its expression is significantly upregulated in various cancers [92,93,94]. HIF-1α likely helps facilitate the metabolic shift from OXPHOS to glycolysis in cancer cells under hypoxic conditions, thereby promoting tumor progression [95]. Several studies have found that HIF-1α regulation, which is facilitated by ncRNAs, plays a large role in glucose metabolism in OSCC. For example, Chen et al. found that in OSCC, miR-199a-5p expression was significantly downregulated, whereas HIF-1α expression was aberrantly upregulated. Both negatively regulate each other [96], with the downregulation of miR-199a-5p expression promoting the upregulation of HIF-1α, which leads to a dramatic increase in GLUT1, HK2, and LDHA protein contents. On the other hand, upregulating HIF-1α expression leads to miR-199a-5p repression, thus forming a dual-regulatory axis wherein they co-enhance glycolysis in OSCC. Furthermore, in HCC, downregulating miR-199a-5p expression can also increase the expression levels of HIF-1α and its downstream genes (i.e., *HK2*), thereby promoting glycolysis [97]. Although the miR-199a-5p/HIF-1α axis appears to be a conserved mechanism driving the Warburg effect across different malignancies, its functional outcomes may be tissue- or context-dependent. In OSCC, this axis is closely associated with hypoxia-induced aggressive phenotypes, including enhanced migration and invasion, suggesting that targeting this pathway may hold specific therapeutic relevance for OSCC progression and metastasis. Collectively, these findings indicate that modulating the miR-199a-5p/HIF-1α axis may represent a rational therapeutic strategy in multiple cancer types, including OSCC. Meanwhile, Shi et al. observed that lncHIFCAR expression was significantly upregulated in both OSCC cell lines (e.g., SAS, HSC-3) and in tumor tissues. When lncHIFCAR binds to HIF-1α, this increases the expression of the target genes of HIF-1α, including *LDHA*, *GLUT1*, and *PDK1*, further promoting glycolysis in OSCC cells [98]. Moreover, lncHIFCAR exhibits prognostic value in OSCC, as lncRNAs are known for their high sensitivity, specificity, stability, and accessibility as biomarkers [98,99]. Thus, regulating the expression of HIF-1α through ncRNAs may be an important mechanism in the treatment of OSCC.

Krüppel-like factors (KLFs) are a group of transcription factors with zinc finger structures, with 18 KLFs identified as key regulators of cell proliferation, differentiation, and apoptosis. Among these, KLF7 plays a significant role in regulating glucose metabolism [100,101]. More specifically, Hei et al. found that circ_0020377 sponges miR-194-5p to upregulate KLF7, which transcriptionally activates HK2 to enhance glycolysis in OSCC cell lines [102]. This suggests that a therapeutic strategy targeting both KLF7 and HK2 could potentially have synergistic anticancer effects, warranting further exploration. Another important regulator of metastasis in OSCC is ZEB1, the main transcription factor that mediates the epithelial–mesenchymal transition [103]. A recent study demonstrated that lncCYTOR regulates glucose metabolism and mitochondrial respiration in OSCC by stabilizing ZEB1 mRNA through an HNRNPC-dependent ubiquitination mechanism [104]. Targeting the KLF7-HK2 axis and ZEB1-mediated pathways may provide novel therapeutic strategies to inhibit glycolysis and metastasis in OSCC.

It is important to recognize that the observed co-upregulation of ncRNAs and glycolysis-related genes in OSCC may not always imply direct regulation. An alternative explanation is chromosomal co-amplification, in which neighboring genes are simultaneously overexpressed due to genomic structural changes. Such events can create transcriptional correlations independent of functional interactions [105], underscoring the necessity of critically evaluating the genomic context of proposed regulatory axes. For example, the genomic loci for the components of the lncPVT1/miR-150-5p/GLUT1 ceRNA network map to three distinct chromosomes: lncPVT1 (8q24.21), miR-150-5p (19q13.33), and GLUT1 (1p34.2) [38]. This physical separation significantly reduces the likelihood that their co-expression is attributable to regional copy-number gain, thereby strengthening the evidence for a functional, post-transcriptional regulatory relationship. Although other confounders like shared transcriptional regulators or technical biases cannot be entirely excluded, chromosomal co-amplification remains the most salient non-functional alternative. It is therefore imperative for future studies to incorporate copy-number profiling to distinguish genuine ncRNA-mediated regulation from chromosomal co-amplification in metabolic control.

## 4. Clinical Implications of ncRNA-Regulated Glycolysis in OSCC

OSCC is an aggressive disease characterized by rapid metastasis, frequent recurrence, and poor prognosis, thus emphasizing the importance of an early diagnosis and timely intervention [106]. Recent studies have demonstrated the potential of ncRNAs as effective biomarkers for disease detection and as therapeutic targets in several cancers [29,32]. Table 1 lists the glycolysis-linked ncRNAs in OSCC with aberrant expression in patient samples. These findings not only highlight the crucial role of ncRNAs in the pathogenesis of OSCC but also emphasize their potential for clinical application.

### 4.1. ncRNAs as Biomarkers in OSCC

Many ncRNAs are differentially expressed in patients with OSCC and healthy individuals, making them valuable diagnostic and prognostic biomarkers for OSCC [109,110]. These ncRNAs that serve as biomarkers for OSCC are primarily obtained from surgically resected tissue, saliva, serum, and exosomes [111,112,113,114]. Surgical biopsy provides a comprehensive view of the tumor microenvironment, whereas liquid biopsy is a non-invasive alternative for detecting ncRNAs [115,116,117]. The diagnostic performance (e.g., sensitivity and specificity) of ncRNAs for OSCC is typically evaluated using receiver operating characteristic (ROC) curves, with the area under the curve (AUC) quantifying overall accuracy [118]. In contrast, the prognostic value of ncRNAs is established through long-term patient monitoring and subsequent survival analyses, such as Kaplan–Meier (KM) curves and multivariate Cox regression [119]. The following section presents several glycolysis-linked ncRNAs with diagnostic and prognostic potential in OSCC.

miR-200c influences the glucose uptake of OSCC cells by downregulating GLUT1 expression [107]. Various studies detecting ncRNAs from multiple sources have all revealed that miR-200c expression levels were significantly reduced in patients with OSCC [120,121,122,123]. From a diagnostic perspective, a study involving 87 OSCC patients found that miR-200c expression was significantly downregulated in tumor tissues compared with adjacent normal tissues (ANT) in 89.7% of patients, and the serum levels of miR-200c decreased progressively with advanced primary tumor stages. ROC curve analysis demonstrated that serum miR-200c could effectively distinguish OSCC patients from healthy controls with an AUC of 0.9481 [107]. For prognosis, Song et al. analyzed a larger retrospective cohort of 204 patients and reported that low miR-200c expression in tumor tissues emerged as an independent prognostic factor for poor recurrence-free survival (RFS) and overall survival (OS), with 3-year rates as low as 36.3% and 52%, respectively [123]. Similarly, miR-218 has demonstrated dual clinical utility across independent cohorts. One study that analyzed serum samples from 68 OSCC patients and 28 healthy controls found that low miR-218 levels could accurately diagnose OSCC, with an AUC of 0.95 [43]. In a two-phase study utilizing tumor tissue from a discovery cohort (n = 58) and a validation cohort (n = 141), Peng et al. demonstrated that each unit decrease in miR-218 expression was independently associated with an approximately twofold increased risk of disease recurrence and death, underscoring its prognostic significance [124].

Instead of regulating glucose transporters, both circBICD2 and circPVT1 target HK2 to promote glycolysis in OSCC [24,59]. Zhao et al. reported that in an independent validation cohort of 90 OSCC patients and 82 healthy controls, salivary circBICD2 levels were significantly elevated in OSCC. This biomarker achieved a diagnostic sensitivity of 74.4%, specificity of 90.2%, and an AUC of 0.863. Furthermore, circBICD2 expression was significantly correlated with the tumor-node-metastasis (TNM) stage and higher tumor grade, suggesting a link to tumor aggressiveness. It also exhibited significant discriminatory power between OSCC and oral leukoplakia (OLK; AUC = 0.819), underscoring its potential clinical utility in the differential diagnosis [125]. Additionally, in a cohort of 50 OSCC patients, circPVT1 expression was significantly upregulated in tumor tissues and showed diagnostic potential with an AUC of 0.787, 68.6% sensitivity, and 86.0% specificity. Elevated circPVT1 expression was also significantly associated with larger tumor size and advanced TNM stage, further supporting its role in disease progression [126].

Although miR-199a-5p and lncHIFCAR both modulate glycolysis in OSCC by regulating HIF-1α, the expression of miR-199a-5p is downregulated while lncHIFCAR is upregulated [96,98]. From a prognostic perspective, Wei et al. analyzed a cohort of 60 OSCC patients and found that low miR-199a-5p expression in tumor tissues was a significant indicator of adverse outcomes [108]. Shih et al. identified lncHIFCAR as a potent oncogenic driver in a cohort of 42 OSCC patients. They showed that high lncHIFCAR expression in tumor tissues was associated with advanced tumor grade and poor differentiation, and that lncHIFCAR itself served as a robust prognostic indicator. KM analysis revealed that patients with high lncHIFCAR expression had significantly worse OS and RFS than those with low expression. Notably, in multivariate Cox regression analysis, high lncHIFCAR expression remained an independent prognostic factor for RFS even after adjusting for conventional clinicopathological parameters. Furthermore, the authors mechanistically linked high lncHIFCAR expression to hypoxia adaptation, providing a biological rationale for its association with aggressive tumor behavior and poor clinical outcomes [98].

Despite the potential of glycolysis-related ncRNAs as OSCC biomarkers, their clinical translation is challenged by substantial tumor heterogeneity and patient variability. As shown in Table 2, different glycolysis-related ncRNAs exhibit distinct associations with diverse clinicopathological characteristics, including tumor stage, lymph node metastasis, differentiation grade, and treatment response. For instance, miR-5787 is specifically linked to cisplatin resistance, while lnc-p23154 is associated with larger tumor size, advanced clinical stage, and lymph node metastasis [23,57]. Moreover, ncRNA expression can be affected by patient-specific factors such as lifestyle. For example, miR-200c levels are related to smoking and tobacco-chewing history, suggesting that individual variations may compromise the reliability of single ncRNA biomarkers [122]. These findings indicate that a single ncRNA is insufficient to reflect the full complexity of OSCC heterogeneity, limiting its utility as a standalone diagnostic or prognostic tool. Therefore, future biomarker research should shift from single indicators to integrative molecular subtyping combining multiple ncRNAs with conventional clinicopathological parameters. Furthermore, although ROC and KM analyses provide preliminary diagnostic and prognostic information, their clinical validity requires rigorous validation in large-scale, multi-center prospective cohort studies.

### 4.2. ncRNAs as Therapeutic Targets in OSCC

Oncogenic ncRNAs are defined as those that promote tumor progression, and these are often upregulated in tumor patients. Conversely, tumor-suppressor ncRNAs, which inhibit tumor progression, are often underexpressed in these patients [127,128]. This suggests that two therapeutic approaches for ncRNAs can be employed in clinical treatment. First is the loss-of-function approach, which aims to competitively inhibit oncogenic ncRNAs by injecting antisense oligonucleotides (ASOs) or locked nucleic acid into the cytoplasm [129,130,131,132]. Moreover, gene editing technology, such as the delivery of the CRISPR/Cas9 system into tumor cells, can be utilized for the knockdown of oncogenic ncRNAs [133,134]. Second is the gain-of-function approach. This involves the chemical modification of the oligonucleotide mimics of tumor-suppressor ncRNAs, which are then administered to upregulate the expression of ncRNAs and inhibit tumor progression [135,136,137,138]. Currently, most studies involve miRNA-based therapies, choosing either the knockdown or overexpression approach depending on the specific expression of ncRNAs. As further research elucidates the mechanisms of lncRNAs and circRNAs in OSCC, their unique therapeutic strategies as miRNA sponges are gradually being explored. It is important to note, however, that a specific ncRNA may exhibit dual roles with opposite effects, acting as either an oncogenic or a tumor-suppressive ncRNA in distinct biological pathways [139,140]. Therefore, it is important to first clarify the specific mechanism of the target ncRNA when exploring ncRNA-targeting therapies.

#### 4.2.1. Tumor-Suppressor ncRNA-Targeting Therapies

In normal cells, specific tumor-suppressor ncRNAs inhibit excessive cell proliferation by restricting the expression of glycolysis-related enzymes, proteins, and transcription factors. This regulatory mechanism suggests that tumor progression in OSCC can be slowed down by restoring the expression of abnormally downregulated tumor-suppressor ncRNAs. Several candidate tumor-suppressor ncRNAs were identified in preclinical studies. These were introduced into OSCC nude mice as mimics, and their therapeutic potential was demonstrated in OSCC cells by modulating the glycolysis pathway.

One of these tumor-suppressor ncRNAs is circ_0000140, which regulates GLUT1 and LDHA expression to suppress glycolysis in OSCC cells through the circ_0000140/miR-182/ cell division cycle 73 (CDC73) axis [141]. Guo et al. demonstrated a significantly reduced tumor growth rate and weight in circ_0000140 overexpression nude mice with OSCC [141]. Additionally, Dong et al. revealed that circ_0000140 can enhance radiosensitivity and decrease the glucose uptake and lactate production of OSCC cells [142]. *In vitro* studies have shown that miR-143 inhibits glycolysis in OSCC by targeting HK2. To further validate its *in vivo* therapeutic effect, Sun et al. injected miR-143 mimics into the tumors of OSCC nude mice, which significantly reduced HK2 expression and LDH activity [54]. Additionally, miR-125a has also been shown to target HK2 in laryngeal squamous cell carcinoma (LSCC), thereby suppressing glycolysis and enhancing radiosensitivity [143]. However, this mechanism has not yet been observed in OSCC. Meanwhile, miR-125b increases the radiosensitivity of OSCC by regulating intercellular adhesion molecule 2 (ICAM2) instead of HK2. Shiiba et al. found that the OSCC cells of miR-125b-transfected groups exhibited a decreased proliferation rate when exposed to radiation compared to negative controls [144]. Further studies should investigate whether miR-125b can similarly target HK2 to modulate radiosensitivity in OSCC. Chen et al. verified that tumors in TSCC nude mice with high miR-5787 expression had a lighter weight, smaller size, and increased cellular apoptosis. Additionally, miR-5787 knockdown also contributed to chemoresistance in TSCC cells, whereas its overexpression partially reversed chemoresistance, potentially through the modulation of OXPHOS and aerobic glycolysis [57]. Collectively, these findings further validate the efficacy of tumor-suppressor ncRNA mimics *in vivo*, demonstrating the clinical potential of gain-of-function strategies and paving the way for therapies targeting glucose metabolism in OSCC.

#### 4.2.2. Oncogenic ncRNA-Targeting Therapies

The abnormal overexpression of oncogenic ncRNAs in OSCC cells accelerates aerobic glycolysis, which promotes their proliferation. Consequently, therapies targeting oncogenic ncRNAs are designed to inhibit or knock out these oncogenic factors, aiming to restore normal cellular glucose metabolism. In animal experiments, several ncRNAs have been demonstrated as effective therapeutic targets for this approach.

Zhu et al. documented that lncCYTOR promotes glycolysis in OSCC via the lncCYTOR–HNRNPC–ZEB1 axis, with its administration leading to a significant decrease in OSCC lung metastasis and metastatic cells *in vivo* [104]. Besides, circBICD2 [24], circMDM2 [20], circNFATC3 [83], circPVT1 [59], and circ_0020377 [102] promote glycolysis in OSCC by functioning as molecular sponges that sequester tumor-suppressive miRNAs. When downregulated, these ncRNAs markedly reduced tumor size and weight in OSCC mouse xenograft models. Furthermore, lncHIFCAR substantially enhances glycolysis in OSCC by acting as a coactivator of HIF-1α. Shih et al. observed a marked decline in the lung colonization of OSCC xenografts and cells after intravenously injecting OSCC cells transfected with sh-HIFCAR into nude mice [98]. In summary, these *in vivo* experiments demonstrate that downregulating oncogenic ncRNAs can inhibit tumor growth and glycolysis in OSCC nude mice, making them potential therapeutic targets.

Although the studies above strongly demonstrate the great potential of glycolysis-related ncRNAs as therapeutic targets for OSCC, most research remains in the preclinical stage, primarily conducted in cell lines and mouse models. The efficacy and safety of these findings in humans require validation through large-scale clinical trials. A key technical challenge in translating these findings into therapies is the efficient and specific delivery of ncRNA mimics or inhibitors to human tumor tissues while avoiding off-target effects and systemic toxicity. Beyond these technical hurdles, a more fundamental challenge is the dual risk of on-target toxicity derived from the complex biology of ncRNAs. First, many ncRNAs exert context-dependent functions, acting either as oncogenes or tumor suppressors in different cancer types [139,140]. Second, numerous candidate therapeutic ncRNAs carry out indispensable physiological functions in normal tissues. For example, miR-143 is critical for regulating retinal neovascularization [145]; miR-125b is essential for normal B-lymphocyte development, but its overexpression can promote leukemogenesis [146]; and circPVT1 modulates cellular senescence, where its knockdown accelerates aging and induces morphological changes such as cell flattening and enlargement [147]. Therefore, systemic therapeutic modulation of ncRNAs must consider both their context-dependent effects and the potential disruption of normal physiological processes.

## 5. Bioinformatics Insights into ncRNA-Mediated Glycolysis in OSCC

### 5.1. Prognostic Impact of Glycolytic Genes in OSCC

**Figure 4:**
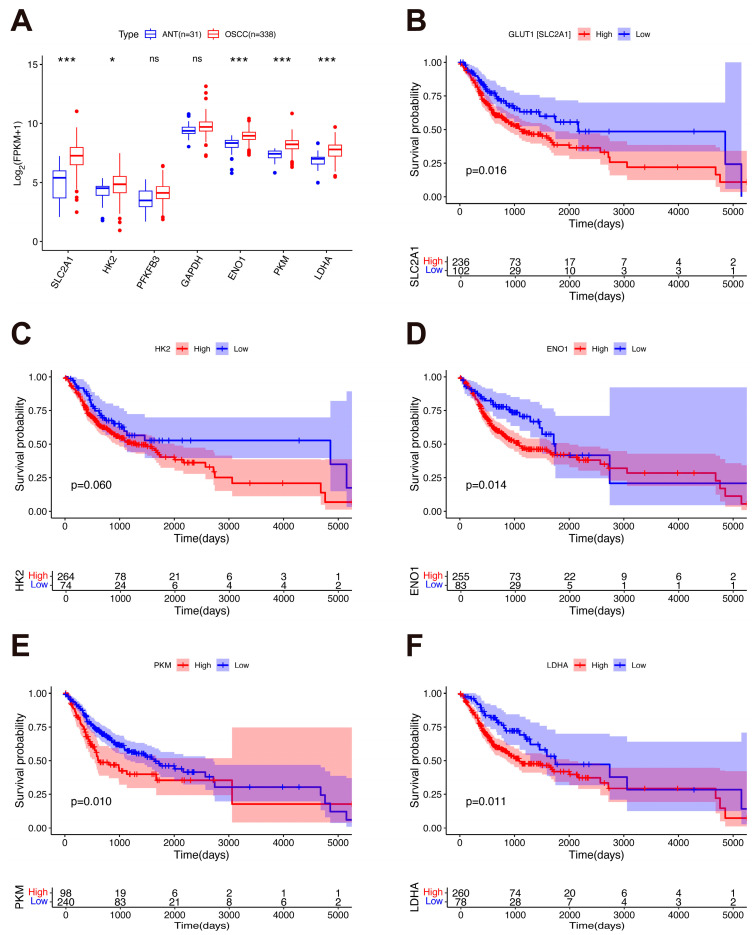
Expression patterns of glycolytic genes and their prognostic impact in OSCC. (**A**) Expression levels of seven classical glycolytic genes (*GLUT1* [also known as *SLC2A1*], *HK2*, *PFKFB3*, *GAPDH*, *ENO1*, *PKM*, and *LDHA*) in OSCC (n = 338) compared to ANT (n = 31). Log_2_(FPKM + 1) was used for the log-scale. Boxplots indicate the median, 25th and 75th percentiles. Whiskers extend to the most extreme values within 1.5 times the interquartile range, and individual points beyond this range are plotted as outliers. Differential expression analysis was performed using the DESeq2 method. ns, not significant. * adjusted *p* < 0.05 and *** adjusted *p* < 0.001. (**B**–**F**) Kaplan–Meier survival curves stratified by high and low expression (based on optimal cutoff values) of glycolytic genes with prognostic significance. Survival differences were evaluated using the log-rank test.

Glycolysis is a central pathway in reprogrammed cancer metabolism, which contributes to tumor progression and poor prognosis [148]. We evaluated the prognostic significance of classical glycolytic genes (*GLUT1* [also known as *SLC2A1*], *HK2*, *PFKFB3*, *GAPDH*, *ENO1*, *PKM*, and *LDHA*) in OSCC by analyzing the RNA-Seq data and clinical information of 369 patients (338 OSCC tissues and 31 ANT) using data from the TCGA database-HNSCC project (Tables S1–S3). Gene expression levels were log-transformed using log_2_(FPKM + 1) for visualization. As shown in Figure 4A, the expression levels of *GLUT1*, *HK2*, *ENO1*, *PKM*, and *LDHA* were significantly upregulated in OSCC tissues versus ANT (adjusted *p* < 0.05). Furthermore, KM survival analysis revealed that patients with high expression of *GLUT1*, *ENO1*, *PKM*, and *LDHA* (but not those with high *HK2* expression) exhibited significantly worse OS compared to those with low expression (*p* < 0.05), highlighting the prognostic value of these glycolytic genes in OSCC (Figure 4B–F).

### 5.2. Screening for Glycolysis-Related ncRNAs with Potential as Biomarkers and Therapeutic Targets in OSCC

**Figure 5:**
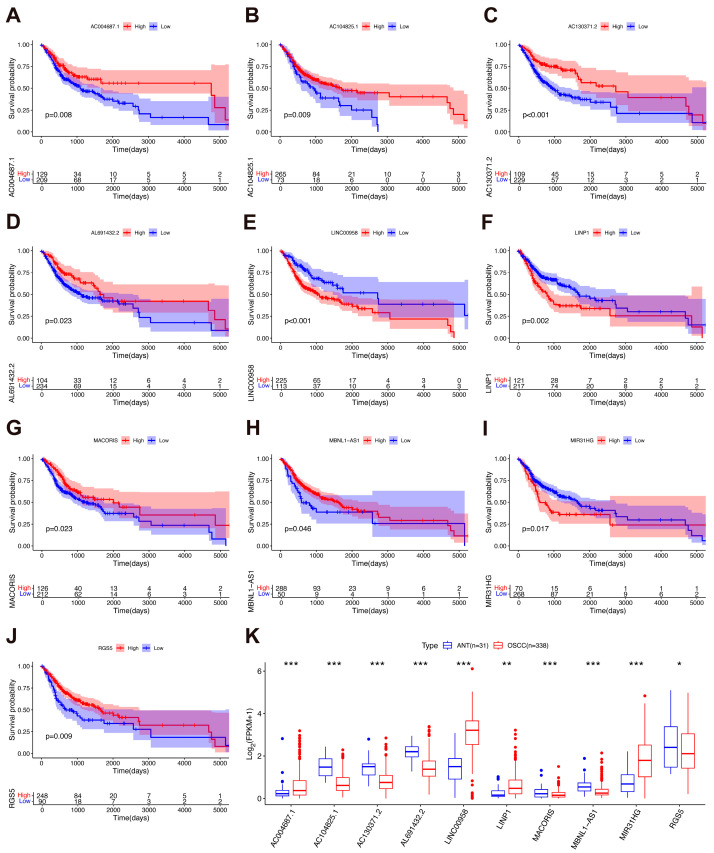
Identification of glycolysis-associated ncRNAs with prognostic potential in OSCC. (**A**–**J**) Survival analysis of 10 glycolysis-related prognostic ncRNAs identified through the screening strategy detailed in Tables S4–S7. High and low expression cohorts were stratified according to the optimal cutoff values. (**K**) Differential expression of candidate ncRNAs between OSCC (n = 338) and ANT (n = 31). Differential expression analysis was performed using raw count data with the DESeq2 method. For visualization, log_2_(FPKM + 1) was used. Boxplots indicate the median, 25th and 75th percentiles. Whiskers extend to the most extreme values within 1.5 times the interquartile range, and individual points beyond this range are plotted as outliers. Differential expression analysis was performed using the DESeq2 method. * adjusted *p* < 0.05, ** adjusted *p* < 0.01, and *** adjusted *p* < 0.001.

To identify glycolysis-related and clinically relevant ncRNAs in OSCC, we designed a screening strategy based on data from the TCGA-HNSCC project. First, we identified 3346 ncRNAs that were differentially expressed between OSCC tissues and ANT (|log_2_FC| > 1 & adjusted *p* < 0.05) (Table S4). After excluding low-abundance transcripts, 317 ncRNAs with FPKM > 0.1 in both OSCC tissues and ANT were subject to further analysis (Table S5). Spearman’s correlation analysis was performed to identify ncRNAs that were significantly associated with key glycolytic genes (*GLUT1*, *HK2*, *ENO1*, *PKM*, and *LDHA*), which yielded 24 glycolysis-related ncRNAs (|r| > 0.4 & *p* < 0.05) (Table S6). After analyzing the KM survival curves, 10 ncRNAs exhibited significant prognostic value (*p* < 0.05) (Figure 5A–J), including 7 tumor-suppressor lncRNAs (AC004687.1, AC104825.1, AC130371.2, AL691432.2, MACORIS, MBNL1-AS1, and RGS5) and 3 oncogenic lncRNAs (LINC00958, LINP1, and MIR31HG) (Table S7). The distinct expression profiles of these candidate ncRNAs suggest their putative involvement in the glycolytic regulation of OSCC (Figure 5K). Further investigations into their mechanisms are needed to validate their regulatory functions in glucose metabolic reprogramming and to evaluate their therapeutic and prognostic potential.

## 6. The Challenges of ncRNAs in Clinical Application of OSCC

### 6.1. Is the Methodology for the Extraction and Detection of ncRNAs Highly Accurate?

Identifying ncRNA signatures through noninvasive methods, such as liquid biopsy, has become more common for diagnosing and prognosticating tumors. However, their clinical application faces significant challenges, primarily due to the low abundance of ncRNAs in body fluids and high interpatient variability [149,150]. The screening and validation of candidate ncRNAs also require large-scale studies of body fluid samples, underscoring the urgent need for standardized protocols in sample extraction and quantification procedures. Further complexities may also arise regarding the selection of appropriate sample sources, variations in sample composition, and the optimization of storage conditions, which are significant barriers to the development of reliable ncRNA-based diagnostic tools. For instance, factors such as dietary habits or oral hygiene may influence the stability of ncRNAs in saliva [151,152], while contamination by hemocytes during extraction can compromise the accuracy of ncRNAs detected in plasma [153]. Other studies have also demonstrated the significant impact of age on the composition of blood ncRNAs [154,155]. Therefore, before ncRNA identification via liquid biopsy becomes the gold standard for diagnosing OSCC, it is first crucial to standardize extraction and detection protocols, improve sample accuracy and stability, and identify OSCC-specific biomarkers. These advancements are essential for liquid biopsy to complement or reduce reliance on tissue biopsy in clinical practice.

### 6.2. Has the Regulatory Mechanism of ncRNAs in Tumor Progression Been Fully Elucidated?

Although many ncRNAs have been identified as potential therapeutic targets, it remains challenging to definitively categorize them as tumor suppressors or oncogenes. This is because of their ability to target multiple genes across diverse signaling pathways. This means that modulating specific ncRNAs may inadvertently affect other pathways and cause unintended effects. Only a few ncRNAs, miR-34a [156,157], miR-143a [158], miR-145a [159,160], miR-20 [161,162], and the let-7 family [163,164], have been well-established as tumor suppressors, whereas miR-21 [165,166], miR-155 [167,168], and miR-181 [169,170] are widely recognized as oncogenic. Due to the limited knowledge regarding their precise downstream targets and regulatory mechanisms, it is challenging to develop ncRNA-based therapies for OSCC that are suitable for clinical trials. For example, lncRNAs and circRNAs, which are miRNA sponges, exhibit unique advantages such as high specificity and stability. This sponge mechanism extends their duration of action and enhances their binding stability, making them attractive candidates for targeted therapy, but *in vivo* studies remain limited. Further research is needed to fully elucidate their mechanisms and optimize the development of ncRNA-based therapies for OSCC.

### 6.3. Can ncRNA Mimics and Inhibitors Maintain Robust Stability and Precise Targeting Efficacy In Vivo?

Despite their therapeutic potential *in vivo*, the clinical application of ncRNAs is hindered by their short half-life, off-target effects, and inefficient delivery. To address these challenges, various delivery strategies such as liposomes, cationic polymers, exosomes, and chemical modifications have been extensively investigated.

Liposomes are the most widely used delivery vehicles for miRNA mimics and inhibitors. For instance, MRX34 is a liposomal miR-34a mimic that has demonstrated anti-tumor efficacy in primary liver cancer, advanced or metastatic tumors, and hematologic malignancies [171,172]. Previously, the *in vivo* delivery of conventional liposomal systems faced several safety issues due to their toxicity, nonspecific cellular uptake, and adverse immune reactions [173]. Fortunately, after continuous optimization of their compositions and structures, the new generation of liposome-based delivery systems has demonstrated substantial improvements in safety, delivery efficiency, and drug loading capacity [174].

Cationic polymers can form polyplexes with RNA fragments, which enhances their interaction with negatively charged cell surface polysaccharides, facilitating transcellular delivery. For example, Fan et al. developed a cationic polylysine-modified cisplatin prodrug that could be combined with a miR-330-3p inhibitor, which exhibited potent anti-OSCC effects [175]. However, despite their high bioavailability and delivery efficiency, these systems can be hindered by interactions with negatively charged serum proteins.

Exosomes have emerged as another promising delivery vehicle, with several advantages such as a long half-life, strong targeting ability, and low toxicity. Exosomes may even exhibit superior antitumor efficacy in pancreatic ductal adenocarcinoma compared to liposomes [176]. Zhang et al. demonstrated that the exosome-mediated delivery of circGDI2 to OSCC cells was able to significantly suppress glycolysis by downregulating GLUT1 and LDHA expression [40]. On the other hand, the challenges of exosome-based therapeutics involve isolating high-purity exosomes and potential immune responses, which are areas for further research.

Finally, the chemical modification of ncRNAs can also enhance the delivery efficiency. The Warburg effect in tumors promotes lactate accumulation, which creates an acidic extracellular microenvironment that facilitates the targeted delivery of pH low insertion peptide (pHLIP)-modified miRNA inhibitors to recipient cells [177].

### 6.4. How Accurate and Specific Are ncRNAs as Biomarkers for OSCC?

The diagnostic accuracy and specificity of individual ncRNAs are currently limited, while research is lacking on multi-ncRNA combinations. Thus, identifying OSCC-specific ncRNA biomarkers remains a significant challenge, and this is crucial for establishing early diagnostic criteria and characterizing the clinical and biological features of OSCC. Fortunately, integrating multiple ncRNA biomarkers into liquid biopsy approaches shows great potential in the development of OSCC-specific diagnostic panels. Piao et al. identified a circulating miRNA panel comprised of miR-92a-3p, miR-92b-3p, miR-320c, and miR-629-5p, which demonstrated robust diagnostic performance with 97.8% specificity and 74% sensitivity in distinguishing patients with OSCC. The serum expression levels of these miRNAs significantly decreased after surgical resection and increased upon recurrence, highlighting their potential for therapeutic monitoring and recurrence surveillance in OSCC [178]. Similarly, Pedersen et al. reported that a combination of miR-30a-5p and miR-769-5p in plasma was able to effectively distinguish patients with OSCC from healthy individuals [179]. Furthermore, Mehterov et al. identified a novel six-miRNA panel (miR-215p, miR-93-5p, miR-133b, miR-146b-5p, miR-155-5p, and miR-182-5p) with diagnostic and prognostic significance in terms of OS [180]. Collectively, these studies highlight the potential of systematically screening and validating multi-ncRNA combinations to improve diagnostic precision and enhance clinical outcomes in OSCC.

### 6.5. How Far Have ncRNA-Based Therapies Advanced from Animal Experiments to Clinical Applications?

For many years, resistance to radiotherapy and chemotherapy has remained a major challenge in the treatment of OSCC. Encouragingly, recent studies have demonstrated that circ_0000140 [141] and miR-125b [144] overexpression was able to enhance radiosensitivity and suppress tumor growth in OSCC mouse models, while miR-5787 [57] overexpression significantly improved cisplatin sensitivity in nude mice with TSCC. Thus, ncRNAs have the potential to resensitize therapy-resistant OSCC cells, providing a direction for the development of combinatory strategies involving ncRNAs and chemotherapeutic drugs. However, a significant gap remains between animal studies and clinical applications. Currently, only a few ncRNAs are under clinical investigation as diagnostic biomarkers for OSCC [5,181,182], while ncRNA-targeting therapies have yet to be reported in patients with OSCC.

Several key challenges hinder clinical translation. Firstly, the high heterogeneity of OSCC driven by HPV status, lifestyle, tumor subsite, and the tumor microenvironment results in inconsistent ncRNA biomarker performance and variable therapeutic responses in preclinical models. Distinct ncRNA profiles between HPV-positive and HPV-negative OSCC reflect divergent oncogenic pathways. For example, miR-125b-5p, miR-214-5p [183], and the TMEM161B-AS1/miR-651-5p axis [184] are specifically dysregulated in HPV-positive tumors, whereas miR-21 [185,186] and miR-155 [186] show no association with HPV status. Notably, this heterogeneity can be further exacerbated by the combined effect of HPV infection and smoking, as this dual exposure is associated with more pronounced dysregulation of specific ncRNAs, such as lncRNAs TMPO-AS1, DDX11-AS1, PCAT-1, and miR-145-5p [187,188]. Dysregulation of these molecules has been linked to poor prognosis in HNSCC. Collectively, this molecular heterogeneity indicates that effective ncRNA-based diagnostics and therapies must be tailored to HPV-defined subtypes. Secondly, substantial disparities exist between preclinical models and human disease. Xenograft models often fail to recapitulate the full complexity of human OSCC, particularly heterogeneity driven by distinct etiologies and lifestyle factors [189]. This mismatch explains why many ncRNA therapeutics effective in animals encounter bottlenecks during clinical translation. Thirdly, effective delivery remains a major hurdle. Beyond stability and off-target effects, local delivery to the oral cavity is further impeded by salivary clearance, mucosal barriers, and enzymatic degradation. To translate ncRNA-based diagnostics and therapies into clinical practice, future studies must systematically overcome these OSCC-specific challenges.

## 7. Conclusions

Glucose metabolic reprogramming is a core hallmark of cancer that enables the uncontrolled proliferation of tumor cells and their adaptation to external stressors such as hypoxia and nutrient deficiency. Accumulating evidence has highlighted the crucial role of ncRNAs, particularly miRNAs, lncRNAs, and circRNAs, in the pathogenesis of OSCC by regulating the expression of glucose transporters, glycolytic enzymes, and transcription factors involved in glucose metabolism. Thus, detecting and modulating ncRNA expression levels can give rise to novel clinical strategies for the diagnosis, treatment, and prognostication of OSCC.

However, several limitations should be considered when interpreting the existing literature. First, many studies in this field had small sample sizes, which may have compromised statistical power. Small cohorts increase the risk of false-positive or false-negative results, hindering reliable validation of the diagnostic and prognostic value of ncRNAs. Second, substantial heterogeneity across studies limits direct comparability and meta-analysis. Variations in ncRNA sources (e.g., tissue, serum, saliva), control selection (ANT vs. healthy donors), detection methods (e.g., qPCR platforms, reference genes), and clinical endpoints all complicate cross-study comparisons. Third, most studies are single-center designs, which may reduce representativeness. Differences in ethnicity, geographic origin, lifestyle, HPV status, and tumor subsite further restrict the generalizability of findings. Therefore, large-scale, multi-center, and prospective studies are urgently needed to validate such ncRNA biomarkers.

Beyond these limitations, a number of research gaps remain to be addressed. It is important to understand how ncRNAs indirectly regulate glucose metabolic reprogramming in OSCC through oncogenic signaling pathways, such as the phosphatidylinositol 3-kinase (PI3K)/Akt pathway. High-throughput sequencing technologies can facilitate the large-scale screening of ncRNA candidates with the potential to become biomarkers or therapeutic targets, as well as aid in elucidating their regulatory networks. In addition to miRNAs, lncRNAs, and circRNAs, other types of ncRNAs implicated in OSCC also warrant further exploration. There is also an urgent need to develop advanced *in vivo* delivery systems that can target specific ncRNAs in OSCC and establish rigorous methodologies to evaluate the safety and efficacy of ncRNA therapeutics. Finally, combining ncRNA-targeting therapies with existing approaches, such as chemotherapy or immunotherapy, could significantly improve OSCC treatment outcomes. Addressing these issues will advance our understanding of ncRNA-mediated glucose metabolism regulation in OSCC and can hopefully facilitate the clinical adoption of ncRNA-targeting therapies.

## Figures and Tables

**Table 1:** Clinical potential of miRNAs, lncRNAs, and circRNAs regulating glycolysis in oral squamous cell carcinoma.

ncRNA	Source	Sample Size and Gender	Study Type	Function	Target	Mechanism	Clinical Significance	Ref.
miR-200c ↓	Tissue	Patient: n = 87 (M/F = 41:46)	Case–control study	Tumor suppressive	GLUT1	miR-200c decreases GLUT1 expression by suppressing Akt phosphorylation, thus inhibiting OSCC glycolysis	Diagnosis, and prognosis	[107]
	Serum	Patient: n = 87 (M/F = 41:46)						
		Control: n = 40						
lnc-p23154 ↑	Tissue	Patient: n = 49 (M/F = 28:21)	Case–control study and animal study	Oncogenic	GLUT1	lnc-p23154/miR-378a/GLUT1	Diagnosis, prognosis and therapeutic target	[23]
		Mouse: n = 5/group (female)				lnc-p23154 increases GLUT1 expression by sponging miR-378a, thus promoting OSCC glycolysis		
lncPVT1 ↑	Tissue	Patient: n = 70 (M/F = 34:36)	Case–control study and animal study	Oncogenic	GLUT1	lncPVT1/miR-150-5p/GLUT1	Diagnosis, prognosis and therapeutic target	[38]
		Mouse: n = 12/group				lncPVT1 increases GLUT1 expression by sponging miR-150-5p, thus promoting OSCC glycolysis		
miR-143 ↓	Tissue	Patient: n = 30	Case–control study and animal study	Tumor suppressive	HK2	miR-143 inhibits glycolysis in OSCC by decreasing HK2 expression	Therapeutic target	[54]
		Mouse: Not specified						
circBICD2 ↑	Tissue	Patient: n = 30 (M/F = 17:13)	Case–control study and animal study	Oncogenic	HK2	circBICD2/miR-107/HK2	Diagnosis and therapeutic target	[24]
		Mouse: n = 20/group (female)				circBICD2 increases HK2 expression by sponging miR-107, thus promoting OSCC glycolysis		
circMDM2 ↑	Tissue	Patient: n = 20	Case–control study and animal study	Oncogenic	HK2	circMDM2/miR-532-3p/HK2	Prognosis and therapeutic target	[20]
		Mouse: n = 10/group (male)				circMDM2 increases HK2 expression by sponging miR-532-3p, thus promoting OSCC glycolysis		
circPVT1 ↑	Tissue	Patient: n = 30	Case–control study and animal study	Oncogenic	HK2	circPVT1/miR-106a-5p/HK2	Therapeutic target	[59]
		Mouse: n = 14/group				circPVT1 increases HK2 expression by sponging miR-106a-5p, thus promoting OSCC glycolysis		
miR-3666 ↓	Tissue	Patient: n = 36 (M/F = 31:5)	Case–control study and animal study	Tumor suppressive	PFKFB3	miR-3666 inhibits glycolysis in OSCC by decreasing PFKFB3 activity	Therapeutic target	[64]
		Mouse: n = 14/group (male)						
lncH19 ↑	Tissue	Patient: n = 6	Case–control study and animal study	Oncogenic	PFKFB3	lncH19/miR-675-5p/PFKFB3	Therapeutic target	[21]
		Mouse: n = 20/group (male)				lncH19-derived miR-675-5p binds PFKFB3 to facilitate glycolysis in OSCC cells		
circNFATC3 ↑	Tissue	Patient: n = 46 (M/F = 30:16)	Case–control study and animal study	Oncogenic	LDHA	circNFATC3/miR-520h/LDHA	Therapeutic target	[83]
		Mouse: n = 12/group				circNFATC3 increases LDHA expression by sponging miR-520h, thus promoting OSCC glycolysis		
miR-199a-5p ↓	Tissue	Patient: n = 60 (M/F = 36:24)	Case–control studies	Tumor suppressive	HIF-1α	miR-199a-5p inhibits glycolysis in OSCC by suppressing HIF-1α expression	Diagnosis and prognosis	[96,108]
lncHIFCAR ↑	Tissue	Patient: n = 15	Case–control study and animal study	Oncogenic	HIF-1α	lncHIFCAR acts as a HIF-1α coactivator, modulates the hypoxia signal pathway and contributes to OSCC progression	Diagnosis, prognosis and therapeutic target	[98]
		Mouse: n = 20/group (female)						
circ_0020377 ↑	Tissue	Patient: n = 68 (M/F = 41:27)	Case–control study and animal study	Oncogenic	KLF7	circ_0020377/miR-194-5p/KLF7	Therapeutic target	[102]
		Mouse: n = 10/group (male)				circ_0020377 increases KLF7 expression by sponging miR-194-5p, thus promoting OSCC glycolysis		

Note: Human tissue controls were obtained from adjacent normal tissues of the corresponding OSCC patients, and serum controls were from healthy individuals. Mouse tissue controls were collected from healthy mice. Upward arrows (↑) indicate upregulation and downward arrows (↓) indicate downregulation. GLUT1, Glucose transporter 1; HIF-1α, Hypoxia-inducible factor 1 alpha; HK2, Hexokinase 2; KLF7, Krüppel-like factor 7; LDHA, Lactate dehydrogenase A; PFKFB3, 6-phosphofructo-2-kinase/fructose-2,6-bisphosphatase 3.

**Table 2:** Associations of glycolysis-related miRNAs, lncRNAs, and circRNAs with clinical characteristics in patients with oral squamous cell carcinoma.

ncRNA	Source	Sample Size and Gender	Clinical Association	Prognostic Value	Ref.
miR-143 ↓	Tissue	n = 30 patients	Associated with tumor metastasis	Not reported	[54]
miR-199a-5p ↓	Tissue	n = 60 patients	Associated with larger tumor size, poor differentiation, lymph node metastasis, and advanced TNM stage	Independent prognostic factor for poor 5-year OS	[108]
miR-200c ↓	Tissue	n = 87 patients (M/F = 41:46)	Associated with primary tumor stage	Not reported	[107]
	Serum	n = 87 patients (M/F = 41:46)	Not reported	Associated with poor OS	
		n = 40 (Control)			
	Tissue	n = 40 patients (M/F = 30:10)	Associated with tobacco chewing/smoking history and poor differentiation	Not reported	[122]
	Tissue	n = 204 patients (M/F = 146:58)	Associated with pN+, advanced TNM stage, and poor differentiation	Independent poor prognostic factor for OS and RFS	[123]
miR-218 ↓	Tissue	n = 68 patients (M/F = 28:30)	Associated with tumor stage	Not reported	[43]
	Serum	n = 68 patients (M/F = 28:30)	Associated with tumor stage	Associated with better OS	
		n = 28 (Control, M/F = 16:12)			
	Tissue	n = 141 patients	Associated with distant metastasis in pN+ patients (2.1-fold increased risk per unit decrease)	Associated with poor DFS and DSS	[124]
miR-5787 ↓	Tissue	n = 126 patients (M/F = 69:57)	Associated with cisplatin resistance	Independent poor prognostic factor for OS	[57]
lnc-p23154 ↑	Tissue	n = 49 patients (M/F = 28:21)	Associated with larger tumor size, advanced clinical stage, and lymph node metastasis	Not reported	[23]
lncCYTOR ↑	Tissue	n = 192 patients	Associated with advanced clinical stage and pathological grade	Associated with poor OS	[104]
lncHIFCAR ↑	Tissue	n = 42 patients	Associated with advanced tumor grade and age	Associated with poor OS and RFS; Independent prognostic factor for RFS	[98]
lncHOXA11-AS ↑	Tissue	n = 16 patients	Associated with lymph node metastasis	Not reported	[68]
lncPVT1 ↑	Tissue	n = 70 patients (M/F = 34:36)	Associated with advanced TNM stage, metastasis, and recurrence	Associated with poor RFS and OS	[38]
circBICD2 ↑	Tissue	n = 30 patients (M/F = 17:13)	Associated with larger tumor size (≥3 cm), advanced TNM stage (III–IV), and lymph node metastasis	Not reported	[24]
	Saliva	n = 90 patients (OSCC)	Associated with advanced TNM stage and tumor grade	Decreased to nearly normal levels post-surgery	[125]
		n = 70 patients (OLK)			
		n = 85 (Control)			
circNFATC3 ↑	Tissue	n = 46 patients	Associated with advanced TNM stage and lymph node metastasis	Associated with poor OS	[83]
circPARD3 ↑	Tissue	n = 47 patients	Associated with advanced clinical stage and lymph node metastasis	Associated with poor OS	[74]
circPVT1 ↑	Tissue	n = 50 patients (M/F = 34:16)	Associated with larger tumor size (≥5 cm) and advanced TNM stage (III–IV)	Not reported	[126]

Note: Tissue controls were obtained from adjacent normal tissues of the corresponding OSCC patients, and saliva/serum controls were from healthy individuals. Upward arrows (↑) indicate upregulation and downward arrows (↓) indicate downregulation. DFS, Disease-free survival; DSS, Disease-specific survival; OS, Overall survival; pN+, Pathological Node-positive; RFS, Recurrence-free survival; TNM, Tumor–Node–Metastasis.

## Data Availability

The original contributions presented in this work are included in the article/Supplementary Materials. Further inquiries can be directed to the corresponding author.

## Supplementary Materials

The following supporting information can be downloaded at: https://www.mdpi.com/article/10.3390/biology15050373/s1, Table S1: Differential expression of glycolytic genes in OSCC tissues vs. ANT; Table S2: FPKM expression matrix of glycolytic genes in OSCC tissues and ANT; Table S3: Differential expression and Kaplan–Meier survival analysis of glycolytic genes in OSCC; Table S4: Differentially expressed ncRNAs in OSCC tissues vs. ANT; Table S5: Expression matrix of the filtered ncRNAs in OSCC tissues (FPKM > 0.1); Table S6: ncRNAs significantly correlated with glycolytic genes (|r| > 0.4, *p* < 0.05); Table S7: Kaplan–Meier survival evaluation of glycolysis-related ncRNAs in OSCC.

## Abbreviations

The following abbreviations are used in this manuscript:

1,3-BPG	1,3-Bisphosphoglycerate
2-PG	2-Phosphoglycerate
3-PG	3-Phosphoglycerate
ADP	Adenosine diphosphate
αKG	α-Ketoglutarate
ALDOA	Aldolase A
ANT	Adjacent normal tissues
ASOs	Antisense oligonucleotides
AUC	Area under the curve
CDC73	Cell division cycle 73
ceRNA	Competing endogenous RNA
circRNAs	Circular RNAs
DFS	Disease-free survival
DHAP	Dihydroxyacetone phosphate
DNMT	DNA methyltransferases
DSS	Disease-specific survival
ENO1	Enolase 1
F-1,6-BP	Fructose-1,6-bisphosphate
F-2,6-BP	Fructose-2,6-bisphosphate
F-6-P	Fructose-6-phosphate
FAD	Flavin adenine dinucleotide
G-3-P	Glyceraldehyde-3-phosphate
G-6-P	Glucose-6-phosphate
G6PD	Glucose-6-phosphate dehydrogenase
GA-3-P	Glyceraldehyde-3-phosphate
GAPDH	Glyceraldehyde-3-phosphate dehydrogenase
GLUT	Glucose transporter
HCC	Hepatocellular carcinoma
HIF-1α	Hypoxia-inducible factor 1 alpha
HK	Hexokinase
HMT	Histone methyltransferases
HNSCC	Head and neck squamous cell carcinoma
ICAM2	Intercellular adhesion molecule 2
IGF2BP3	Insulin-like growth factor 2 mRNA binding protein 3
KLFs	Krüppel-like factors
KM	Kaplan–Meier
LDHA	Lactate dehydrogenase A
lincRNAs	Long intergenic ncRNAs
lncRNAs	Long non-coding RNAs
LSCC	Laryngeal squamous cell carcinoma
m^6^A	N^6^-methyladenosine
MCT	Monocarboxylate transporter
miRNAs	MicroRNAs
MT-CO3	Mitochondrial cytochrome c oxidase subunit 3
NAD	Nicotinamide adenine dinucleotide
NADH	Nicotinamide adenine dinucleotide hydride
NADPH	Nicotinamide adenine dinucleotide phosphate
ncRNAs	Non-coding RNAs
NQO1	NAD(P)H quinone oxidoreductase 1
OS	Overall survival
OLK	Oral leukoplakia
OSCC	Oral squamous cell carcinoma
OXPHOS	Oxidative phosphorylation
PDH	Pyruvate dehydrogenase
PDK-1	Pyruvate dehydrogenase kinase 1
PEP	Phosphoenolpyruvate
PFK	Phosphofructokinase
PFKFB	6-Phosphofructo-2-kinase/Fructose-2,6-bisphosphatase
PGAM	Phosphoglycerate mutase
PGI	Glucose-6-phosphate isomerase
PGK	Phosphoglycerate kinase
pHLIP	pH low insertion peptide
pN+	Pathological Node-positive
PI3K	Phosphatidylinositol 3-kinase
piRNAs	PIWI-interacting RNAs
PK	Pyruvate kinase
PKM2	Pyruvate kinase M2
PPP	Pentose phosphate pathway
RBPs	RNA-binding proteins
RFS	Recurrence-free survival
RIP	RNA immunoprecipitation
RISC	RNA-induced silencing complex
ROC	Receiver operating characteristic
RNA Pol II	RNA polymerase II
rRNAs	Ribosomal RNAs
SIX1	Sine oculis homeobox homolog 1
siRNAs	Small interfering RNAs
snRNAs	Small nuclear RNAs
snoRNAs	Small nucleolar RNAs
TCA	Tricarboxylic acid
TCGA	The Cancer Genome Atlas
TF	Transcription factor
TME	Tumor microenvironment
TNM	Tumor-node-metastasis
TSCC	Tongue squamous cell carcinoma
UTR	Untranslated region
